# Precise and error-prone CRISPR-directed gene editing activity in human CD34+ cells varies widely among patient samples

**DOI:** 10.1038/s41434-020-00192-z

**Published:** 2020-09-01

**Authors:** Shirin R. Modarai, Sambee Kanda, Kevin Bloh, Lynn M. Opdenaker, Eric B. Kmiec

**Affiliations:** 1 Gene Editing Institute, Helen F. Graham Cancer Center & Research Institute, Newark, DE USA; 2 The Cawley Center for Translational Cancer Research, Newark, DE USA

**Keywords:** DNA damage and repair, Haematopoietic stem cells

## Abstract

Clustered Regularly Interspaced Short Palindromic Repeats (CRISPR) and their associated CRISPR-associated nucleases (Cas) are among the most promising technologies for the treatment of hemoglobinopathies including Sickle Cell Disease (SCD). We are only beginning to identify the molecular variables that influence the specificity and the efficiency of CRISPR- directed gene editing, including the position of the cleavage site and the inherent variability among patient samples selected for CRISPR-directed gene editing. Here, we target the beta globin gene in human CD34+ cells to assess the impact of these two variables and find that both contribute to the global diversity of genetic outcomes. Our study demonstrates a unique genetic profile of indels that is generated based on where along the beta globin gene attempts are made to correct the SCD single base mutation. Interestingly, even within the same patient sample, the location of where along the beta globin gene the DNA is cut, HDR activity varies widely. Our data establish a framework upon which realistic protocols inform strategies for gene editing for SCD overcoming the practical hurdles that often impede clinical success.

## Introduction

Sickle Cell Disease (SCD) is a severe hereditary form of anemia caused by a single nucleotide mutation altering the sixth codon of the beta globin gene (HBB) [1]. There is now renewed optimism for the development of gene therapy because novel genome editing technologies have emerged [2]. CRISPR-directed gene editing is being used to either disable suppressive elements upstream from the start of the fetal globin gene or to correct the well-known mutation in the defective beta globin gene [1]. Currently, a Phase 1/2 clinical trial for sickle cell disease examining safety and efficacy of autologous CRISPR-Cas9 edited CD34+ cells (ClinicalTrials.gov; NCT03745287) is in the recruitment phase [3]. The approach is to isolate cells from patients, edit the CD34+ cells at the erythroid lineage-specific enhancer region of the BCL11A gene, and then re-introduce the modified cells back into the same patient. While reawakening silenced genes is a clever and logical approach, ultimately one goal of any gene editing strategy should be to repair the single base mutation responsible for the disorder.

Investigators have begun to coalesce around an RNP particle consisting of a specific guide (sg)RNA complexed with a bacterial nuclease, most often Cas9 [4–6] as the preferred editing tool. It is now known that the RNP can be effectively delivered to progenitor cells ex vivo and in combination with a single-stranded donor DNA template, can execute single nucleotide correction [7, 8]. The single-stranded DNA donor can be generated by replication and expression of single-stranded DNA from Adeno-associated virus template [9] or synthetic single-stranded DNA oligonucleotides (ssODNs) [10–12]. Both types of molecules have their advantages and disadvantages. Viral vectors have a long, yet controversial history as therapeutic agents [13] and ssODNs while readily scalable must be delivered to the target tissue with high efficiency to enable the gene correction event.

Our lab has become interested in defining foundational variables that have an overarching influence on the success of CRISPR-directed gene editing in human cells. We have taken a decidedly reductionist approach in patient progenitor cells by examining the efficiency of point mutation repair, indel formation, and on-site mutagenesis [14, 15]. We sought to examine how the diversity of patent samples impacts the efficacy of gene editing at a given target site, in this case beta globin. This type of variability is likely not controllable but must be appreciated before CRISPR/Cas achieves widespread use.

In this manuscript, we execute genome alteration in CD34+ HSPCs from a variety of patients targeting two unique sites for gene editing of beta globin. Our data suggest that CD34+ cells support CRISPR-directed gene editing and on-site mutagenesis at different frequencies with both outcomes appearing to be influenced by the source of human cells used in each experiment. The genetic diversity has a dramatic impact on the outcome. In addition, we screen for changes in p53 levels and find that its expression varies as a function of the source of the CD34+ cells. Our data demonstrate that genetic diversity among patient samples regulates the efficiency and precision of homology directed repair in human CD34+ cells.

## Materials and methods

### Cell culture

Bone marrow CD34+ cells (12 different sources of CD34+ cells each obtained from an individual de-identified donor) were purchased from Stem Express. Additional information is included about each donor in Supplementary Table S1. For simplicity, each donor is referred to as a different patient (i.e., patient 1 is from one donor and noted in this study as P1). Cells were cultured in suspension and maintained at 0.5–1.0 × 10^6^ cells/ml and incubated at 37 °C and 5% CO_2_. All CD34+ cells were grown in specific culture media and conditions as previously described [15].

### Assembly of CRISPR/Cas9 RNP complex

The TrueCut Cas9 Protein V2 was purchased from Invitrogen (catalog no. A36499) and the single guide RNAs (sgRNAs) for both the G5 and G10 cut sites were ordered through Synthego. The sgRNA/Cas9 individual components were incubated at room temperature for 15 min to form the complete CRISPR/Cas9 RNP prior to Nucleofection.

### Optimization of the delivery of CRISPR/Cas9 RNP complex and ssODNs

We varied the sgRNA concentrations from 4–8 µg, Cas9 concentrations from 7.5–15 µg, and ssODNs from 1.35–8.1 µg in 2.0 × 10^5^ CD34+ cells and used program ER-100 on the Lonza 4D nucleofector. The Lonza P3 Primary cell 4D-Nucleofector X-Kit was used for these experiments. The optimized RNP condition and ssODN used in all the experiments in the manuscript was 8 µg sgRNA to 15 µg Cas9, which is a 2:1 pmol ratio of sgRNA to Cas9. In addition, a 5.4 µg concentration of the 72-mer ssODN was the optimal concentration. Preliminary work showed optimization of nucleofection kit on a variety of CD34+ cell sources (i.e., donors) based on the pmax GFP plasmid uptake analyzed by flow cytometry. From the optimized parameters of the nucleofector and Lonza kit, the optimal transfection efficiency was chosen, and the same protocol was used for subsequent CD34+ targeted samples.

### Gene editing reactions

The targeted CD34+ cells were grown in the culture for 72 h, harvested and genomic DNA was isolated for sequencing. The sequencing data was analyzed for damage assessment using the software program, Tracking of Indels by DEcomposition (TIDE; Netherlands Cancer Institute, https://tide.nki.nl/) [16] and the sequencing data were also analyzed for percent HDR (Sickle Cell mutation) using the software program, Tracking of Indels (TIDER) [17]. CD34+ whole cell populations were analyzed by TIDE and TIDER, for wild type cells, mock transfected cells, and CRISPR/Cas9 RNP transfected cells.

### DNA sequencing and mutagenesis analysis

PCR was performed using Amplitaq Gold Fast PCR 2X master mix (Thermofisher). Sanger sequencing was performed using the SeqStudio Genetic Analyzer (Applied Biosystems). After PCR amplification using the HBB primers [15], the PCR product was cleaned using Qiaquick PCR purification kit (Qiagen) and Big Dye Terminator PCR was performed using Big Dye Terminator v3.1 (Thermofisher). PCR products were purified once more using the Big Dye Xterminator kit (Thermofisher) and then sequenced.

### Flow cytometric analysis of p53

Targeted cells were fixed in 4% paraformaldehyde with 0.1% Triton-X, for 30 min at room temperature and washed twice with PBS. Cells were incubated in a blocking buffer (10% goat serum, 0.2% BSA and PBS) with a p53 (D0-7) primary mouse monoclonal antibody (Cell Signaling Technology, catalog # 48818) at a 1:50 dilution, and compared to cells incubated with primary mouse monoclonal IgG Isotype control (Cell Signaling Technology, catalog # 53484) at the same concentration as the primary antibody. Samples were incubated for 1 h at room temperature and then washed twice. A goat anti-mouse Alexa Fluor 488 secondary antibody, at a 1:200 dilution, was added to all samples and incubated for one hour at room temperature. After two washes with PBS, samples were resuspended in 500 µl PBS and put through the BD LSRFortessa analyzer. All data were collected off the BD FACS Diva software and analyzed on FCS Express 6 software.

### Statistical analysis

For statistical comparisons, Student’s *t* test was used where appropriate.

## Results

### Schematic of CRISPR ribonucleoprotein and overall workflow of this study

**Fig. 1:**
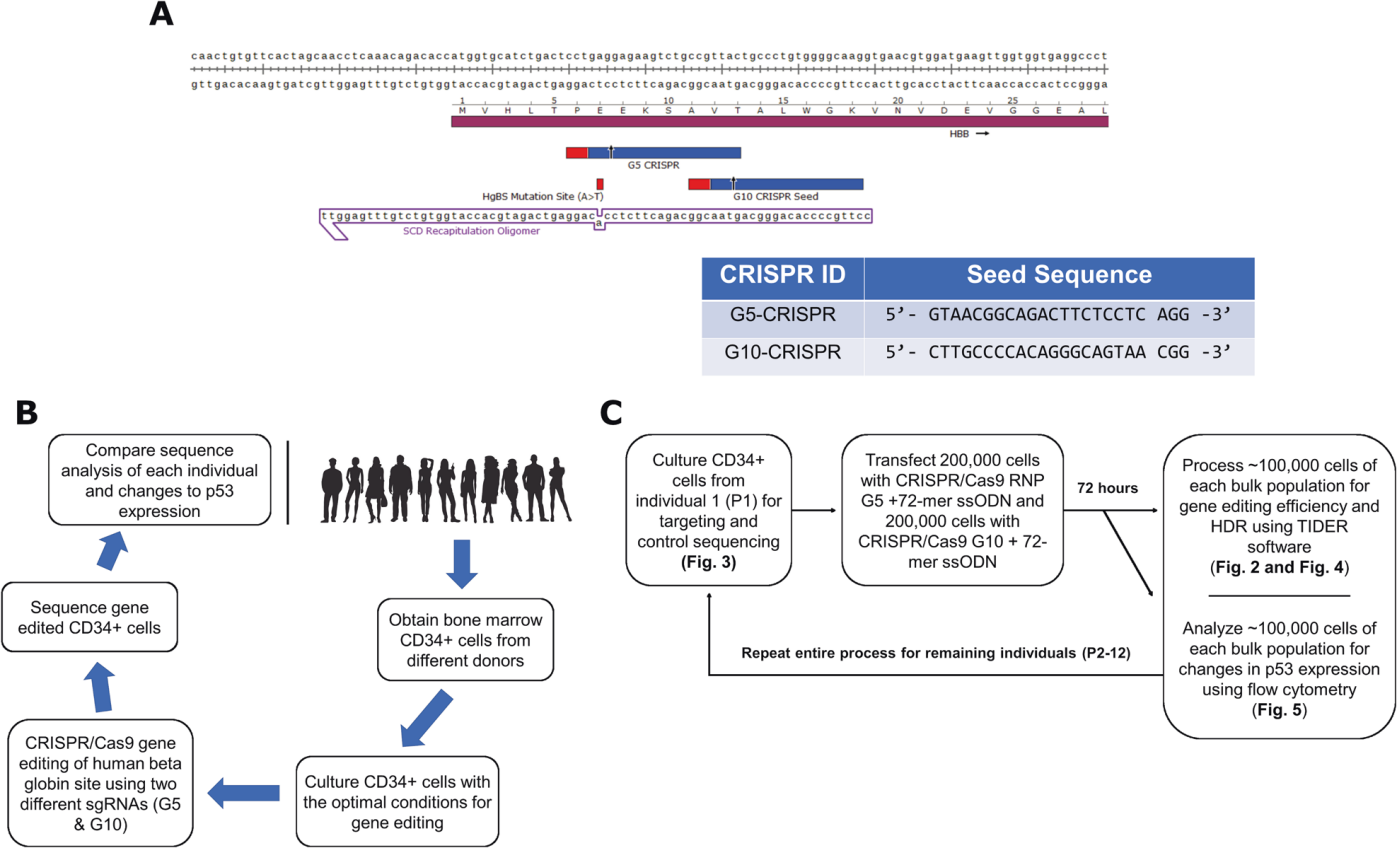
Overview of experimental design and CRISPR/Cas9 RNPs used for this study. **a** Schematic of the cleavage sites, G5 and G10, for the human beta globin gene and seed sequences for the crRNAs. Also shown is the 72-mer ssODN sequence that contains the single base pair exchange to create a mismatch to produce the sickle cell sequence. The oligonucleotide used in these experiments is 72 bases in length, bearing phosphorothioate-modified linkages at the three terminal bases. **b** This panel represents the overall workflow of how each donor’s CD34+ cells were treated and analyzed for this study. A total of 12 different donor’s CD34+ cells were obtained and analyzed. Of note, the use of the word donor and patient is interchangeable for the samples in this study, as donor 1 is also patient 1 or P1 for short. **c** This panel represents a more detailed schematic of the workflow done for each sample of CD34+ cells that were analyzed in this study.

Recent reports suggest that the RNP catalyzes high levels of gene editing activity at the beta globin locus [7, 8, 18] and in some cases, executes HDR with minimal indel formation [19]. The schematic in Fig. 1a depicts the sequence for the HBB gene and the *G5* CRISPR and *G10* CRISPR seed sequences used in this study [7]. Our own research has confirmed high levels of activity of these two HBB sites using plasmid and RNPs [15]. HDR reactions were initiated within CD34+ cells with a 72-mer ssODN to direct single base exchange. The schematic shown in Fig. 1b outlines the general workflow of this manuscript, and more details in Fig. 1c.

### CRISPR-directed gene editing at the beta globin gene catalyzed by a ribonucleoprotein complex and a single-stranded DNA donor template

**Fig. 2:**
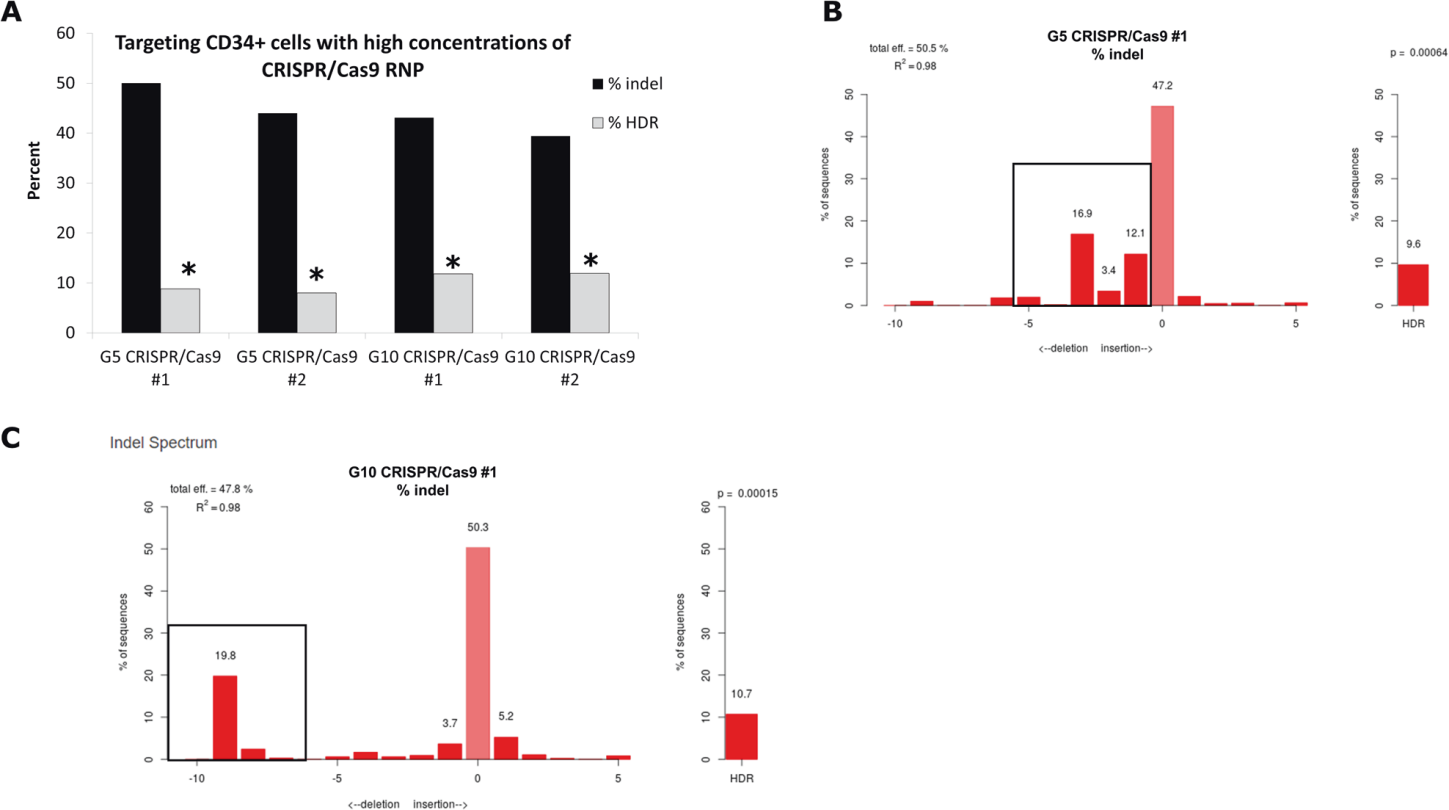
Nucleofection of CD34 + cells using different CRISPR/Cas9 RNPs. **a** Cells targeted with 8 µg G5 sgRNA/15 µg Cas9 RNP and 5.4 µg of 72-mer ssODN and analyzed with TIDER software for percent indels and HDR. This concentration of CRISPR/Cas9 and ssODN was found to be the optimal concentrations, in our hands, for targeting the CD34+ cells with significant HDR. The graph represents data from two experiments targeting the same source of CD34+ cells with CRISPR/Cas9 with the G5 sgRNA (#1 and #2 are independent experiments), and two experiments targeting the same CD34+ cells with CRIPSR/Cas9 with the G10 sgRNA (#1 and #2 for each experimental set). **b, c** Representative TIDER plots of indel and HDR activity catalyzed by the G5 sgRNA and G10 sgRNA CRISPR/Cas9 (experimental set #1 for each CRISPR/Cas9). The majority of the indels are 5 bp deletion or less when using CRISPR/Cas9 close to the mutation site, as can be seen by the black box (**b**), while there are larger sized indels when using the CRISPR/Cas9 that cuts further away from the HBB mutation site. The data presented in this figure are from patient 8 CD34+ cells that were targeted using both the G5 and G10 CRISPR/Cas9 RNPs and 72-mer ssODN, in duplicate reactions.

Our results indicate that the genomic target in most of the cells has been altered as demonstrated by the presence of insertions and/or deletions (Fig. 2a). In many cases, we observe significant levels of HDR coupled to a significant amount of indel formation, on-site mutagenesis. Figure 2a exhibits data from one patient sample targeted with the G5 and G10 CRISPR RNPs, respectively. The first set represents experiments carried out with the G5 CRISPR-complex and the second set represents experiments carried out with the G10 CRISPR-complex. We continued the analyzes by developing an indel profile, outlining the type of insertions or deletions created by the action of the G5 or G10 complex in each reaction mixture (Fig. 2b, c). The population of indels (deletions) formed by the G5 complex averages ~3 bases (framed within the black boxes, Fig. 2b). In contrast, the spectrum of indels created by the action of the G10 complex is >5 bases (framed within the black boxes, Fig. 2c).

### Sequence variation among different sources of CD34+ cells reveals a unique indel pattern formation based on where the target sequence of the CRISPR/Cas9 RNP

**Fig. 3:**
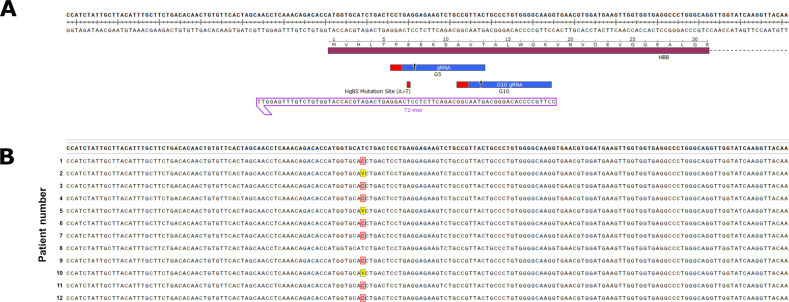
The wild-type sequencing of each patient’s CD34+ cells prior to nucleofection with CRISPR/Cas9 RNPs. **a** Schematic of the cleavage sites, G5 and G10, for the beta globin gene and where they each cut in relation to the sickle cell mutation. In addition, the HDR 72-mer oligo sequence is shown for point of reference to where the seed sequences cut on the beta globin gene. **b** Cells from each patient were collected as a reference for what the individual’s DNA sequence was prior to CRISPR/Cas9 RNP targeting. Every patient had similar nucleotide sequences near the beta globin site, expect for a single nucleotide polymorphism (SNP) found in patients 2, 5, and 10, denoted by a Y, and that could be a cytosine or thymine that location.

The DNA sequence at the beta globin gene is known to contain several polymorphisms and thus we thought it prudent to sequence the targeted region. Figure 3a displays the beta globin gene and the two specific CRISPR/Cas9 cut sites generated by seed sequences G5 and G10. The sequence files represent the wild-type sequence of each patient’s CD34+ cells, covering a span of around 200 base pairs, including where the sickle cell mutation site is, and in our case, there is no sickle mutation in all the donor sequences we analyzed (Fig. 3b). Patients P2, P5, and P10 harbor the well-known single nucleotide polymorphism SNP is rs334 (http://useast.ensembl.org/index.html). Additional sequencing, 740 base pairs upstream from the mutation site and 1033 base pairs downstream from the mutation site, indicated no genetic variations on or around the beta globin gene (data not shown). Some changes in the wild-type sequencing was observed in some of the patient samples (over 500 base pairs upstream from the cut site), but due to their location, we anticipated no change to CRISPR/Cas9 targeting [20, 21].

**Fig. 4:**
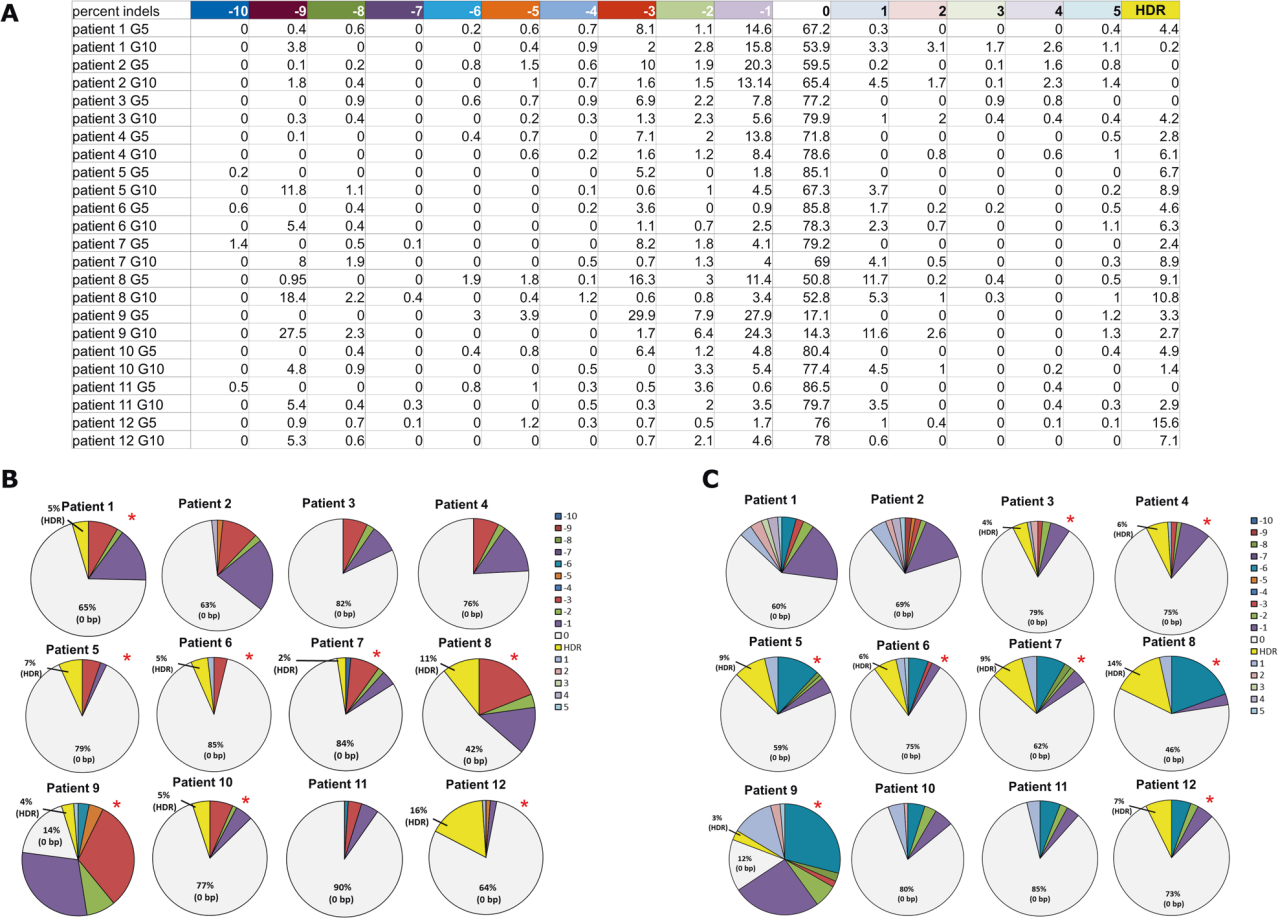
Nucleofection of CD34+ cells using different CRISPR/Cas9 RNPs. **a** The table represents the total indel pattern and value output from TIDER software analysis for each of the 12 patient samples targeted with both G5 and G10 CRISPR/Cas9 RNPs. **b** Cells targeted with 8 µg G5 sgRNA/15 µg Cas9 with 5.4 µg of 72-mer ssODN and analyzed with TIDER for percent indels and HDR. The pie charts represent the total indel pattern that resulted from each patient’s experimental targeting. The 0 bp percentages represent the combined value of the total amount of sequence that did not equal any insertions or deletions (gray area of pie charts), as well as any HDR reactions (yellow area of pie charts). **c** Cells targeted with 8 µg G10 sgRNA/15 µg Cas9 with 5.4 µg of 72-mer ssODN and analyzed with TIDER for percent indels and HDR. The pie charts represent the total indel pattern that resulted from each patient’s experimental targeting. The 0 bp percentages represent the combined value of the total amount of sequence that did not equal any insertions or deletions (gray area of pie charts), as well as any HDR reactions (yellow area of pie charts). All values shown in the pie chart are indels that were scored 1% or higher on TIDER software analysis. The total percentages of indels are shown fully in panel A. All nucleofection reactions were performed with the same number of CD34+ cells, same nucleofection parameters and same time of analysis. For each panel, an *N* = 12 (12 different individuals) was analyzed. All pie charts with red asterisks means a significant HDR reaction as occurred, and a colored key is present in each figure panel to correspond to the indel sizes.

CD34+ cells from 12 different patients were propagated under identical optimized conditions and analyzed for HDR activity and indel formation. The de-identified patient samples are designated patient 1, 2, 3 etc. in the G5/G10 CRISPR complex used in the targeting experiments is indicated on the right side of the patient number (Fig. 4a). The gene editing activity is presented for each patient sample. HDR is measured by the percent of cells in the population exhibiting the specific base change (A → T) and corresponding cells within the population bearing the indicated indel pattern are also displayed. Figure 4b, c provides a visual representation of gene editing activity deemed significant and equal to or more than one percent of the cell population. Figure 4b represents each patient’s cells that were targeted with the G5 CRISPR/Cas9 RNP and 72-mer ssODN, using the same standard conditions and concentrations for all samples. The same procedure was used for Fig. 4c, except the G10 CRISPR/Cas9 RNP and 72-mer ssODN combination was used. The data reveal significant differences in the efficiency of HDR activity within each population. In addition, categories of indels varies among samples including cases where insertions are present without deletions and vice versa. Interestingly, HDR distribution and efficiencies appear to be similar when the same population is treated with either G5 or G10. For both G5 and G10 CRISPR RNP reactions, there is significant differences in the degree of HDR and the types of indel patterns. When targeting the beta globin gene using the G5 CRIPSR/Cas9 RNP, there are smaller sized indels that include −1, −2, and −3 base pairs. Interestingly, when targeting the same gene using the G10 CRISPR/Cas9 RNP, there are deletions that are more than −5 base pair deletions and more insertions overall than with the G5 CRISPR/Cas9 RNP targeted cells. These data confirm our original gene editing profiles presented in Fig. 2. However, activity in patient 12 did not reveal −1 base pair and −3 base pair deletions as observed in the other eleven patient’s CD34 + cells, targeted with the G5 CRISPR RNP. Patients 2, 3, and 4 whose CD34+ cells were targeted with the G10 CRISPR RNP did not exhibit the same larger sized −8 to −9 base pair deletions.

### Differential expression of total p53 catalyzed by a ribonucleoprotein complex and a single-stranded DNA donor template as a measure of off-target mutagenesis

**Fig. 5:**
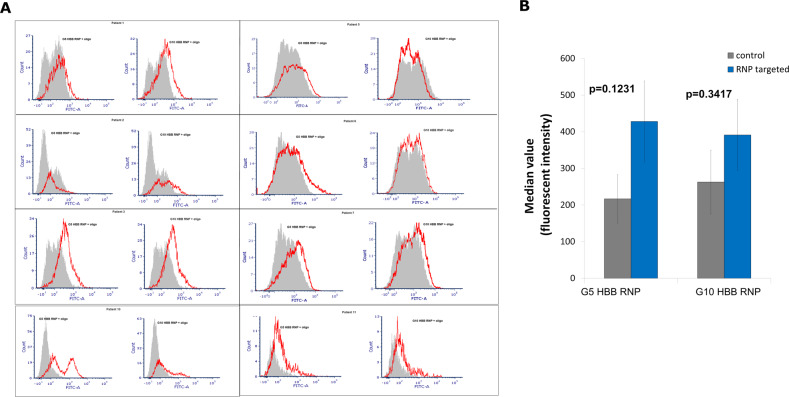
p53 expression in CRISPR-targeted CD34+ cells. Approximately 100,000 CD34+ cells that were targeted with either the G5 CRISPR/Cas 9 RNP + 72-mer ssODN, or G10 CRISPR/Cas9 RNP+ 72-mer ssODN were used for p53 analysis. All cells were collected 72 h post nucleofection and analyzed for p53 expression. The samples were fixed and stained for p53 and the gating of the histograms for all samples were set against an appropriate IgG antibody control. **a** The gray shaded histograms represent the mock control samples which are the cells treated with the appropriate CRISPR RNP and ssODN, but no nucleofection. It is important to note that mock nucleofector samples were also run as another control and there was no difference seen between any of the controls. The red lined histograms represent the targeted cells with the appropriate CRISPR RNP and ssODN. The samples were analyzed on the FITC channel on the BD LSRFortessa. **b** The graph represents the average percent positive p53 expression from eight of the patient samples that were analyzed in this study. The remaining four that were not analyzed, either did not tolerate the CRISPR/Cas9 RNP targeting or not enough cells survived post-nucleofection to performs both sequencing analysis and p53 expression. The graph shows that the average values, of all eight samples, between the control cells and the targeted cells. There seems to be no significant change in p53 expression when the CRISPR/Cas9 RNP targeting was employed. The error bars represent the S.E.M.

Recent reports suggest that CRISPR-directed gene editing in progenitor cells can lead to an increase in expression levels and/or an activation of the tumor suppressor gene, p53 [22–25]. Since we have been examining the differential response of CD34+ cells from a diverse patient population, we asked whether levels of p53 change in response to gene editing activity catalyzed by either of the CRISPR/Cas complexes. We are aware that electroporation and perhaps nuclear affection can affect p53 expression during the first hours after DNA introduction [26]. For our experiments, we processed half of the cells from targeted experiments described in Fig. 4 and performed flow cytometric analysis to look for the changes in total p53 protein expression after the influence of electroporation has been minimized. The Histogram profiles in panel A reveal the percent shift in FITC, which measures the percentage of positive p53 cells (Fig. 5a, b). The gene edited cells were stained with a primary antibody that recognizes intracellular p53 protein levels. The histogram plots all show the control cells (gray, shaded histogram) compared to the targeted cell population (red, outlined histogram). Figure 5a reveals variability among patient samples with minimal change in p53 expression (Patient 2) or significant increases (Patient 10). There is some difference in the presence of p53 expression based on where the targeting occurs within the beta globin gene, although there is variability within patient samples targeted with either G5 or G10. Figure 5b represents the average mean intensity values of the eight patient samples analyzed for p53 expression, and that value does not reflect a significant change from the control cells.

## Discussion

Of the many inherited diseases amenable to gene editing, SCD holds a certain fascination for workers in the field since a solution, single-base correction, seems logical and attainable [27]. While impressive levels of HDR and genetic conversion have been reported, they are usually not robust, perhaps due to the heterogeneous population of cells and the reaction conditions used in the targeting experiments.

For studies that focus on the correction of the sickle cell mutation, most research groups choose the RNP complex to deliver the payload [7, 8, 28, 29]. We too observe the most consistent levels of indel formation and significant HDR when the RNP is part of the reaction [15]. Even when the level of HDR is found to be significant, the position of the cleavage site relative to the targeted base generates mutagenic footprints. The results are informative as they indicate that on-site proximal and distal mutagenesis appears to be dependent on the position of the CRISPR/Cas9 cut site [7, 30]. Despite the position and extent of indels, once again, we have not been able to eliminate indel formation. We utilized the TIDER software program to profile the population of precisely repaired genetic targets and those were error-prone repair has taken place. When the comparable software program, CRIS.py, is used to analyze data, the size and quantity of indels generated in gene editing reactions are nearly identical (Supplementary Fig. S1). Occasionally, some variation in the number of precise of HDR events can be seen in data generated by NGS.

We believe the genetic heterogeneity causes outcome variance as the results outlined in Fig. 3 and Supplementary Table S1 are generated from different patients. Elegant work from Lessard et al. [31], revealed that human genetic variation within the patient’s genome modulates on-site targeting and influences off-site mutagenesis. These data suggest that patient variance is significant and could lead to the failure of CRISPR-directed gene editing in clinical applications. Furthermore, such genetic variability has already been examined in some detail by Murphy et al. in obtaining and analyzing progenitor cell samples from four different haplotypes [32]. Their work indicated a highly differential response to CRISPR-directed gene editing based on haplotype and on the extent of cell differentiation. Furthermore, Bradley et al. demonstrated a more sensitive method to identify not only off-target effects, but also large deletions that cause on-site mutagenesis [33]. Human pluripotent stem cells can acquire p53 mutations as a result of CRIPSR/Cas9 gene editing and thus, p53 levels in each patient sample should be monitored [34].

DNA damage and replication stress are known to stabilize and activate p53 to direct phosphorylation of downstream signaling pathways [35]. In our studies, we predictably observe wide-ranging variability in p53 expression levels that align in some cases with the distribution of indels observed at the genetic level. More work is needed to establish robust correlations between the degree of genetic damage and the levels of p53 activation, but, at the very least, p53 should be monitored routinely in cells selected in a gene editing protocol [23]. While our data do not specifically show any correlation with p53 levels and percent of indels, more importantly HDR, the relationship between p53 mediated DNA damage response along with CRISPR/Cas9 gene editing is important to analyze in future studies. We also recognize that four patient samples were not analyzed for p53 expression because they either failed to survive or expand enough post-nucleofection to analyze for both indels and HDR along with p53 expression, perhaps highlighting a limitation of this study.

In this report, we demonstrate that patient samples vary widely in their capacity to execute CRISPR-directed gene editing at both the cellular and genomic levels, an inherent variability that must be considered when clinical protocols are developed, and clinical expectations are set (Supplementary Table S2). While we achieved a significant level of indels and HDR in this report, other ways to enhance the efficiency are always improving. For correcting the SCD mutation, the alternative use of viral HDR donors has been found to increase HDR since it provides a continuous amount of donor DNA, so combined with optimal nucleofection conditions, there may be improvements in SC mutation repair percentages [9]. The use high-fidelity Cas9 variants or other nucleases, such as Cas12a, in some cases has been shown to increase HDR over Cas-nucleases [36, 37]. We are expanding our efforts in this area so that we can continue to contribute information toward a more robust clinical protocol for the use of CRISPR- directed gene editing as a treatment for SCD.

## Supplementary information

Supplementary table 1

Supplementary table 2

Supplementary figure 1

Supplementary figure and table legends
