# Supplementary material for: Precise and error-prone CRISPR-directed gene editing activity in human CD34+ cells varies widely among patient samples
Source: Gene Ther. 2020 Sep 1;28(1):105–13. doi: 10.1038/s41434-020-00192-z (PMC7902267; doi:10.1038/s41434-020-00192-z)
Supplement: Supplementary file 1 — Supplementary table 1 [file 41434_2020_192_MOESM1_ESM.pptx]

## Slide 1
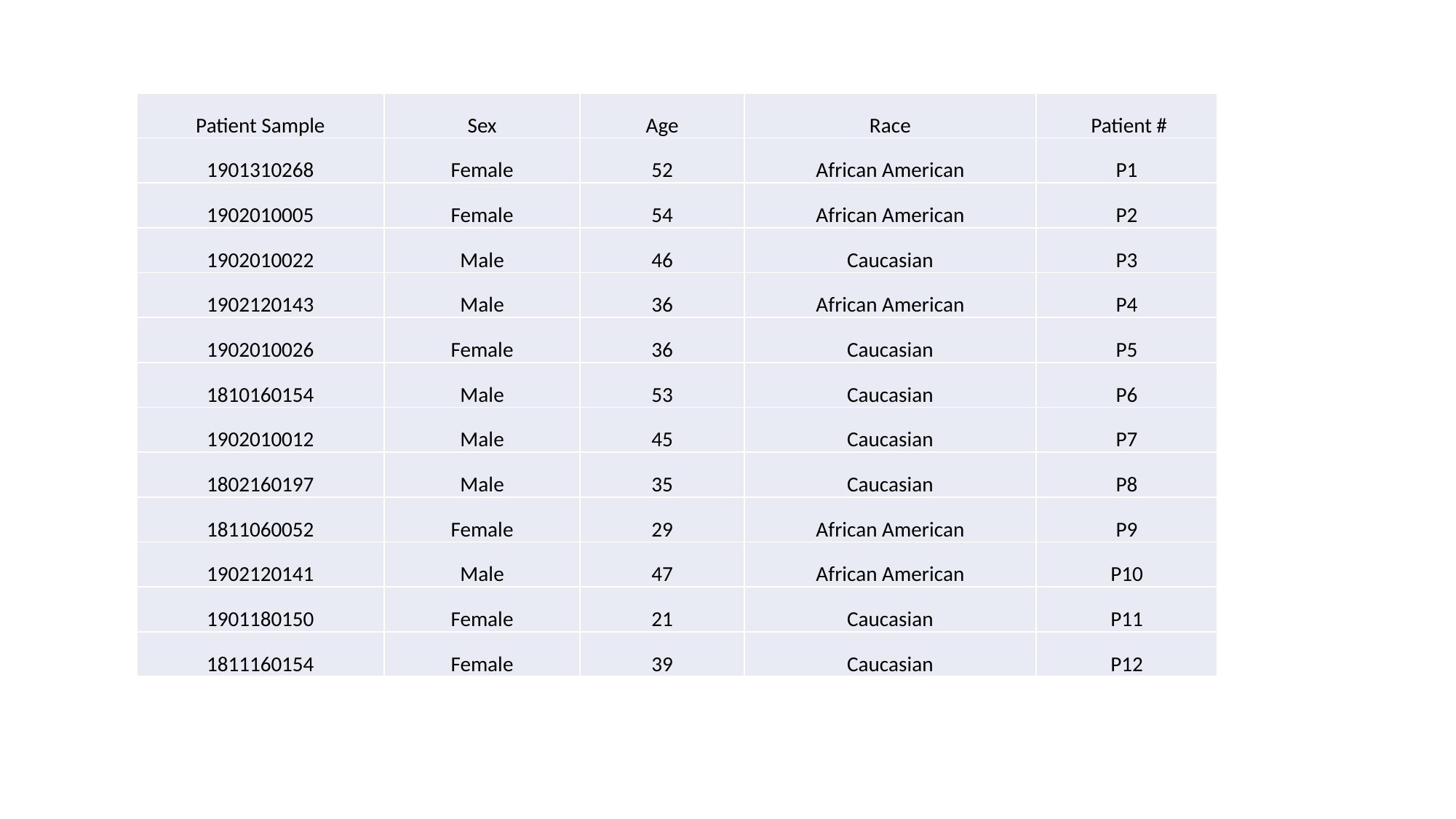

| Patient Sample | Sex | Age | Race | Patient # |
| --- | --- | --- | --- | --- |
| 1901310268 | Female | 52 | African American | P1 |
| 1902010005 | Female | 54 | African American | P2 |
| 1902010022 | Male | 46 | Caucasian | P3 |
| 1902120143 | Male | 36 | African American | P4 |
| 1902010026 | Female | 36 | Caucasian | P5 |
| 1810160154 | Male | 53 | Caucasian | P6 |
| 1902010012 | Male | 45 | Caucasian | P7 |
| 1802160197 | Male | 35 | Caucasian | P8 |
| 1811060052 | Female | 29 | African American | P9 |
| 1902120141 | Male | 47 | African American | P10 |
| 1901180150 | Female | 21 | Caucasian | P11 |
| 1811160154 | Female | 39 | Caucasian | P12 |
