# Supplementary material for: Precise and error-prone CRISPR-directed gene editing activity in human CD34+ cells varies widely among patient samples
Source: Gene Ther. 2020 Sep 1;28(1):105–13. doi: 10.1038/s41434-020-00192-z (PMC7902267; doi:10.1038/s41434-020-00192-z)
Supplement: Supplementary file 2 — Supplementary table 2 [file 41434_2020_192_MOESM2_ESM.pptx]

## Slide 1
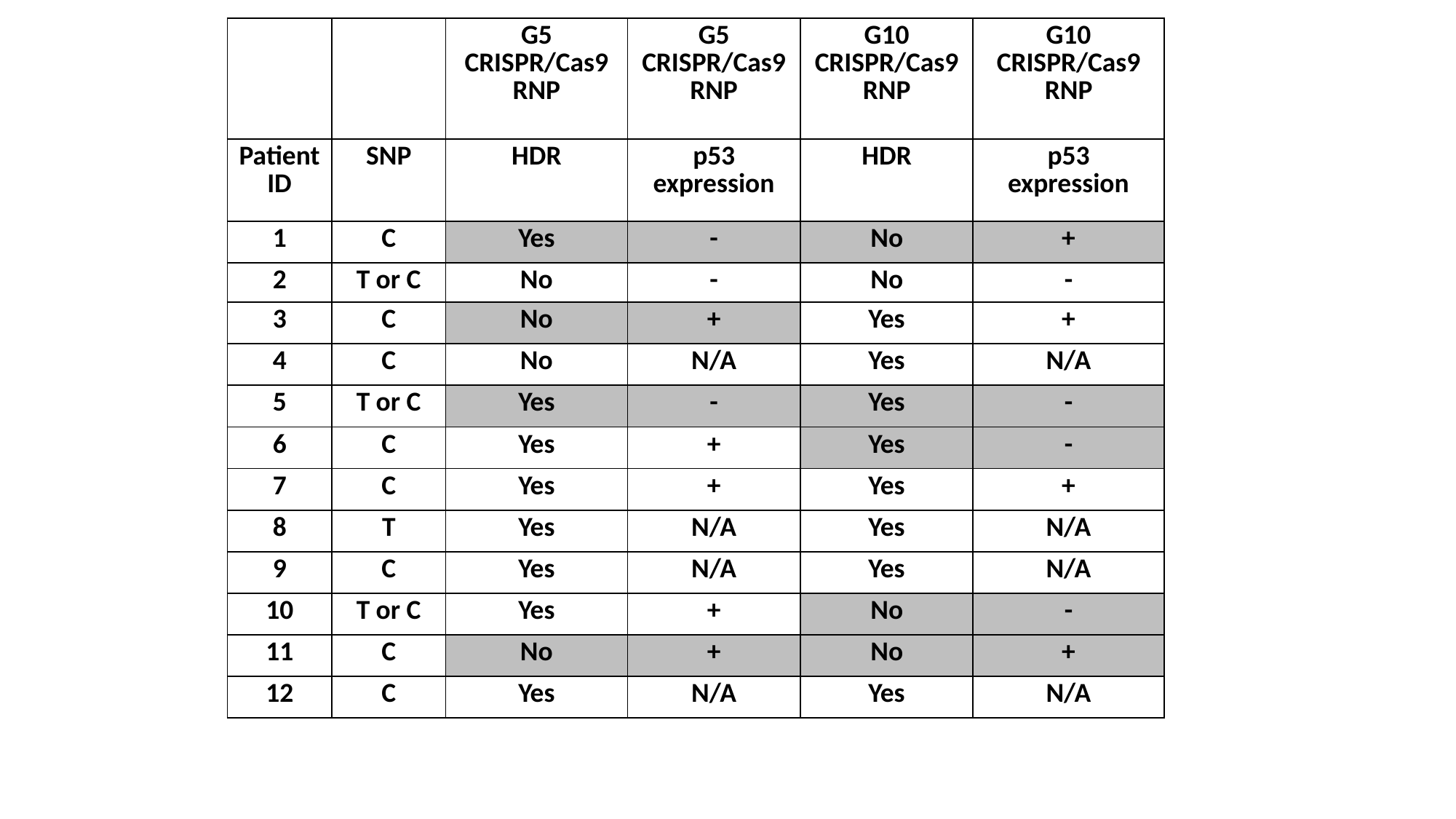

| | | G5 CRISPR/Cas9 RNP | G5 CRISPR/Cas9 RNP | G10 CRISPR/Cas9 RNP | G10 CRISPR/Cas9 RNP |
| --- | --- | --- | --- | --- | --- |
| Patient ID | SNP | HDR | p53 expression | HDR | p53 expression |
| 1 | C | Yes | - | No | + |
| 2 | T or C | No | - | No | - |
| 3 | C | No | + | Yes | + |
| 4 | C | No | N/A | Yes | N/A |
| 5 | T or C | Yes | - | Yes | - |
| 6 | C | Yes | + | Yes | - |
| 7 | C | Yes | + | Yes | + |
| 8 | T | Yes | N/A | Yes | N/A |
| 9 | C | Yes | N/A | Yes | N/A |
| 10 | T or C | Yes | + | No | - |
| 11 | C | No | + | No | + |
| 12 | C | Yes | N/A | Yes | N/A |
