# Supplementary figures and images for: Precise and error-prone CRISPR-directed gene editing activity in human CD34+ cells varies widely among patient samples
Source: Gene Ther. 2020 Sep 1;28(1):105–13. doi: 10.1038/s41434-020-00192-z (PMC7902267; doi:10.1038/s41434-020-00192-z)

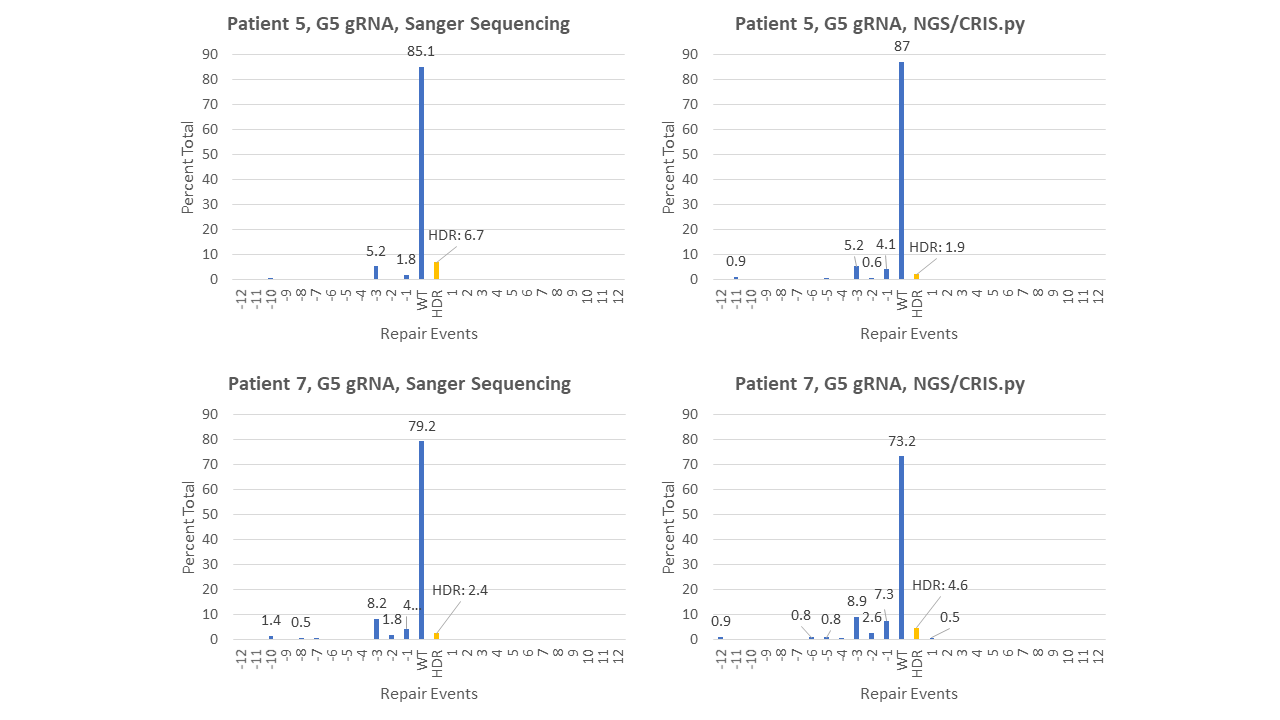

Supplement: Supplementary file 3 — Supplementary figure 1 [file 41434_2020_192_MOESM3_ESM.tif]
