# Supplementary material for: Precise and error-prone CRISPR-directed gene editing activity in human CD34+ cells varies widely among patient samples
Source: Gene Ther. 2020 Sep 1;28(1):105–13. doi: 10.1038/s41434-020-00192-z (PMC7902267; doi:10.1038/s41434-020-00192-z)
Supplement: Supplementary file 4 — Supplementary figure and table legends [file 41434_2020_192_MOESM4_ESM.docx]

**Supplemental Table 1.** **Sources of CD34+ cells used in these experiments.** Twelve different lots of CD34+ cells (different donors) were purchased from StemExpress. The range of different ages, race, and sex is indicated. All donor’s demographic is provided from StemExpress and no other identifying information of the donor is provided.

**Supplemental Table 2.** **Summary of study results.** From our investigation, there seems to be no conclusive correlation that can be drawn from our data that ties the specific SNP observed in these patient samples to the efficiency of HDR or pattern of p53 expression. For example, patient’s 2, 5 and 10 all have the same SNP, yet only patient 5 exhibits significant HDR and no change in p53. Patient sample 2 is negative for any HDR or p53 expression, and patient 10 exhibits mixed results. Additionally, patient 8 is the only one with no SNP at that location of the DNA sequence, yet also exhibits significant HDR. On the other hand, patient 9 also exhibits significant HDR and indels, yet has a different SNP than patient 8. It is important to note that there is no p53 data available for patients 4, 8, 9, and 12 due to limitations on total cell numbers and *in vitro* cell expansion, post-targeting. The remaining 8 patients all have analysis done on experiments including overall HDR efficiency and p53 expression, for both the G5 and G10 CRISRP/Cas9 RNPs. Interestingly, of all the samples that were available for TIDER analysis and p53 analysis, 10 of them followed the trend that has been reported in the literature where hard to transfect cells exhibit transient inhibition of p53 post-nucleofection or electroporation, and this improves overall HDR and efficiency of genome editing^38,39^. While we don’t inhibit p53 in our study, it seems there is no change in p53 expression from the control cells in cases where the CRISPR/Cas9 RNP and ssODN transfection resulted in significant HDR. Further studies would need to be done to see if inhibiting p53 would result in a similar HDR efficiency. In addition, out of the 16 remaining possible sample combinations, 9 of them (highlighted gray) seem to follow the trend of no p53 expression along with significant HDR, and vice versa. When these 10 samples exhibit significant HDR, there is no increase in p53 expression (i.e. patient 5). However, in some of our sample cases, when there is no significant HDR, there is a significant increase in p53 (i.e. patient 11). It is interesting we observe this reported finding to some degree, but a larger sample size study for future research would help support these results in gene editing efficiencies of stem cells. Taken together, there may be a link between p53 expression, HDR efficiencies and certain SNPs in the beta globin gene, but no conclusive statements can be made in regard to HDR and the common SNP that is located within 10 base pairs of the SCD mutation site we see in our analysis.

**Supplementary Figure 1. Targeting experiment results comparing Sanger sequencing and NGS.** Supplementary Figure 1 displays indel rates and HDR efficiencies for Patient 5 and Patient 7 when targeted with the G5 CRISPR/Cas9 RNP, via both Sanger sequencing and NGS.  The Sanger data was analyzed using TIDER^17^, while the NGS data was analyzed using CRIS.py^40^.  While rare indels differ between the two analyses, overall indel distributions and HDR rates between the two are highly similar.  Due to the higher precision with which NGS data can be parsed and analyzed, the HDR rates reported via CRIS.py would be considered more accurate.
